# Foot and Mouth Disease

**Published:** 1884-04

**Authors:** 


### THE FOOT AND MOUTH DISEASE.

THIS damaging, though not fatal, pest seems to be acquiring an alarming degree of extension in this country at the present time. Numerous theories have been started as to how it got among the Western cattle. There is but one true cause, however, *and that is the utter futility of the United States system of quarantine for imported cattle as now carried out.*

*The Foot and Mouth Disease has walked into this country under the eyes of the United States Quarantine Officers and in no other way has it got here.* There are things called quarantine stations, but they are no more such than any private farm is.

The United States Cattle Commission does not seem to know what the word quarantine means, or how to apply it to imported cattle. To quarantine cattle, or anything else, means that it is to be looked upon as diseased, or that a contagio-infectious disease is possible to appear among them at any time, until the period fixed by law has elapsed; or, in other words, the animals should be subjected strictly to quarantine regulations from the time and place of their arrival until the quarantine is over.

This is not done! They are treated in a most careless manner. They can be visited by most any body at any time.

I have just returned from Portland, Maine, where I visited the so-called United States Quarantine, passing over the road *over which the infected cattle were driven, contrary to law, from the steamer on which they arrived.*

On no place on this road is there a sign to indicate that it is dangerous, though the local authorities have forbidden cattle to be driven over it, and I may here state that Dr. Geo. H. Bailey has acted in a most creditable manner in this matter; all places where native cattle are infected being indicated by appropriate signs, and entrance forbidden. How is it with the United States grounds? There is a lane about half a mile long leading to them. Neither at its entrance nor any where on it is there a single sign indicating that infected cattle have been driven over it, nor is there any sign on the buildings of the station to indicate what they are, or what the animals are diseased with, or to forbid entrance.

It is a miserable old wreck of a barn. Not seeing any one round, *I went in and looked over the cattle, and only as I drove away did a woman appear, and ask me "if I had a pass, and tell me I ought to have had one in order to have gone in."* And this is a United States Quarantine Station, controlled by the Veterinary Commission of the Treasury!

It is no wonder that the stock-raising interests complain. If the work of the Bureau for Animal Industry is to be of this nature we do not want it. It can be a valuable department of our Government, however.

I will not touch upon this subject further at present, as I intend to write an article upon it for our next issue.

The Government wants Veterinary advisers who know their business, not empirics or uneducated men, or selfish pettifoggers, such as they now have on their Commission. What government, but ours, on earth would appoint an uneducated, non-graduated empiric on such a Commission?

What government, but ours, would retain any man on such a Commission after such a blunder as was made at Portland?

